# Cultural adaptation and validation of the Sidamic version of the World Health Organization Quality-of-Life-Bref Scale measuring the quality of life of women with severe preeclampsia in southern Ethiopia, 2020

**DOI:** 10.1186/s12955-021-01872-z

**Published:** 2021-10-12

**Authors:** Birhanu Jikamo, Mulat Adefris, Telake Azale, Kassahun Alemu

**Affiliations:** 1 Department of Epidemiology and Biostatistics, Institute of Public Health, College of Medicine and Health Sciences, University of Gondar, Gondar, Ethiopia; 2 Department of Gynecology and Obstetrics, Gondar University Hospital, College of Medicine and Health Sciences, University of Gondar, Gondar, Ethiopia; 3 Department of Health Education and Behavioral Sciences, Institute of Public Health, College of Medicine and Health Sciences, University of Gondar, Gondar, Ethiopia; 4 School of Public Health, College of Medicine and Health Sciences, Hawassa University, Hawassa, Ethiopia

**Keywords:** Translations, Cultural adaptation, Reliability, Validation, Exploratory factor analysis

## Abstract

**Background:**

Women with severe preeclampsia often present with more health complaints compared to those with uncomplicated pregnancies. Estimating the quality of life of women affected with severe preeclampsia could provide direction for further interventions. However, the current measurement of the quality of life has not been culturally adapted and validated for this population. This study aimed to translate, culturally adapt, and test the reliability and validity of the World Health Organization Quality-of-Life-Bref Scale (WHOQOL-BREF) in southern Ethiopia among women with severe preeclampsia.

**Methods:**

An institutional-based cross-sectional study was conducted in southern Ethiopia in selected hospitals with randomly recruited women with severe preeclampsia. Cultural adaptation and validation techniques were used to translate and adapt the WHOQOL-BREF scale. Face, content validity, forward and backward translations, and synthesis were computed using an expert panel. The scale was pretested and adjusted accordingly. Internal consistency (Cronbach’s alpha) and test–retest reliability (Intraclass Correlation Coefficient = ICC) were examined. Confirmatory factor analysis (CFA) was computed to test the fit of the structure to the local setting before conducting exploratory factor analysis (EFA). Multiple methods for determining the number of factors extracted (scree test, eigenvalues) were used. We compared the original English structure with the new structure in the study setting and extracted a new structure using EFA.

**Results:**

The internal consistency reliabilities ranged from 0.8045 to 0.9123 indicating good-to-excellent reliability. The item‑level content validity ranged from 0.86 to 1.00; the scale‑level content validity index was 0.97. In CFA, the model fit indices were unacceptable (Comparative Fit Index (CFI = 0.87), Root Mean Square Error of Approximation (RMSEA = 0.23), Standardized Root Mean Square Residual (SRMR = 0.38), Tucker Lewis Index (TLI = 0.85) and (PCLOSE = 0.00). Three new factor structures were extracted using EFA for current research with a total variance was 91%.

**Conclusions:**

The failure of the original scale in this study population highlights the importance of culturally adapting tool to local settings. EFA confirmed a three-factor structure, inconsistent with the original English structure.

**Supplementary Information:**

The online version contains supplementary material available at 10.1186/s12955-021-01872-z.

## Background

In low income countries, pregnant women’s’ mental, physical, physiological processes, and quality of life (QoL) have not been studied [1]. Predisposing factors that contribute to pregnant women’s’ vulnerability to mental disorders include sleep disturbances, re-experiencing delivery time, and anxiety [2]. Severe preeclampsia is also a stressor with risk factors for the occurrence of physical, social, and mental disturbances [3]. Women with severe preeclampsia frequently have reported physical complaints in the pregnancy and the postpartum period including headache, right upper quadrant pain, visual disturbances, loss of attention, concentration, and fatigue [4].

Many studies have identified the risk of severe preeclampsia on maternal QoL [5–8]. Woman with severe preeclampsia have presented with serious mental distress compared with normotensive women [5]. The severity of mental and psychological diseases has increased when the early onset of the disease was < 30 weeks [7, 8]. Women with a history of severe preeclampsia had more cognitive impairment later in life than those with normotensive women [5].

Some studies have compared the performances of tools for assessing maternal QoL [9–12]. WHOQOL-BREF tool is reliable and valid for measuring maternal QoL [10]. The European Quality of Life Scale-five dimension (EQ-5D) is a tool that is used to detect significant differences in individual health status [12]. However, it has been criticized for having poor sensitivity to improvements in health conditions associated with low morbidity and being unable to detect small changes in health situations [9]. The Short Form Survey 36-item (SF-36) tool is a generic tool used to measure health-related QoL; however, it does not incorporate preferences into its scoring procedure [10]. Another tool derived from the (SF-36) is the Short-Form Six-Dimension (SF-6D) tool used to measure the preference-based measure of health [10]. However, the tool is known to over-predict the value of the poorest health conditions and may not be sensitive to changes in conditions of high morbidity [10]. Because disease-specific QoL tools do not allow for cross-disease comparison, the literature suggested that the World Health Organization Quality-of-Life-Bref Scale (WHOQOL-BREF) is a generic health-related QoL tool that could be useful for any disease condition and to compare with other diseases conditions [13]. Compared to the EQ-5D or SF-6D tools the WHOQOL-BREF tool has very strong cross-cultural applicability and is thus readily suitable for culturally diverse contexts [9, 10].

The WHOQOL-BREF tool was developed originally in English and was proposed for use in English-speaking countries [14, 15]. However, using this tool in non-English-speaking countries has been linked to inaccurate and unreliable estimates [15]. Numerous studies have highlighted the importance of validation of the WHOQOL-BREF tool in non-English-Speaking settings [16–23]. A Norwegian study found that an acceptable internal consistency of the physical, psychological and environmental domains [16]. A study from Iran indicated that the WHOQOL-BREF tool had an acceptable degree of internal consistency in measuring the QoL health condition [19]. The WHOQOL-BREF tool has a good internal consistency, construct, and discriminant validity for any populations having any health conditions [20].

Some studies have identified the validation of the WHOQOL-BREF tool in Ethiopia [14, 17]. However, previously validated tools are not always valid in different settings, cultures, or contexts, possibly due to poor translation [24]. Furthermore, findings based on such tools may not accurately reflect what they are intended to measure. It is necessary to use a locally validated tool that has also been assessed to ensure it can measure the QoL of women with severe preeclampsia in a specific setting to provide accurate and reliable estimates [5, 24].

Sidamigna is one of the widely spoken languages in southern Ethiopia [25, 26]. Compared with other languages in southern Ethiopia it is the primary spoken mother tongue languages of 19.6% of people in Sidama, 10.5% in Wolayita, 8% in Hadiya, 7.1% in Gurage, 6.9% in Gamo, 5.4% in Kafa, and 4.1% in Amharic [25, 26]. In southern Ethiopia, many of the pregnant women attending outpatient clinics do not understand official and/or English language. Therefore, translation of the WHOQOL-BREF tool into the Sidamic language would increase its utility among this population. A validated tool, translated into the Sidamic local language, could be used in similar study populations in Ethiopia’s Sidamic cultures. Therefore, this study aimed to translate, culturally adapt and test the reliability and validity of the WHOQOL-BREF when measuring the quality of life of women with severe preeclampsia in southern Ethiopia.

## Methods

### Study design and setting

An institutional-based cross-sectional study was conducted in Sidama zone, southern Ethiopia in September 2019 in two government primary hospitals, Leku and Yaye hospital. The 2019 population of the zone was 3,893,817 [27]. There are thirteen hospitals in the zone, and 128,650 pregnant women were eligible for antenatal care (ANC) in 2019. Of these, 107,841 pregnant women attended ≥ 4 ANC visits. In 2019, 94,172 women gave birth by skilled birth attendants. Of these, 1,231 women gave births using cesarean delivery [27].

*Translation process and pilot test* (see in method sections in Additional file 1).

### Full psychometric test

Women with severe preeclampsia who delivered within the two Government primary hospitals were included in the study. Those critically ill and unable to respond were excluded from the study. A simple random sampling technique was used to recruit women with severe preeclampsia in the delivery room. The number of participants per parameter was considered for sample size estimation in the psychometric analysis [28]. The ratio of 10:1 participants per parameter was used for sample size estimation for factor analysis [28]. The estimated sample size was 240. However, a non-response rate of 10% was assumed. Of those 264 women with severe preeclampsia who were included for the full Psychometric test.

### Data collection procedures

A pre-tested and locally translated tool was used for data collection. The client's medical records were retrieved for each participant as a mode of delivery for index child, the reason for cesarean section if the mode of delivery using cesarean section, danger signs, and symptoms, previous history, and management of women with severe preeclampsia, current maternal, and prenatal outcomes. A face-to-face interview was conducted using home-to-home visits.

The tool comprised socio-demographic and economic characteristics, previous maternal factors, neonatal characteristics, the locally translated WHOQOL-BREF quality of life scale, and medical records. The training was given for the data collectors, and they were midwives in the profession.

Severe preeclampsia was defined as a single record of BP ≥ 160 and DBP ≥ 110 mmHg, ≥ 20 weeks of gestational age, with confirmed proteinuria, and one or more signs of headache, blurred vision, epigastric pain, and vomiting [29].

### Data quality assessment

Two days of training were given for data collectors on how to collect data from medical records and face-to-face interviews. Each item was coded from the four domains of the WHOQOL-BREF tool had been coded as missing due to the domain score calculated by substituting a person’s specific average across the completed items in the same tool. For example, if a respondent does not have a value for item D21, how much do you enjoy life? In the psychological domain, but has answered all of the other items in that domain, then the value for item D21 would be the average of the remaining five items.

### Data processing and analysis

Descriptive statistics were used to characterize the study participants in terms of socio-demographic and obstetric variables. To identify the variation of the true scores, the standard error of measurement was tested [29]. Test–retest was analyzed using the IBM Statistical Package for the Social Sciences (SPSS), Version 23. Data from EFA was conducted using AMOS 23.0 and STATA Version 14 was used for maximum-likelihood estimation.

Content validity index was computed at the item level (I-CVI) and scale level (S-CVI). There were three methods to calculate S-CVI, but the averaging calculation (S-CVA/Ave) method was preferred and used. Seven experts, the I-CVI of 0.78 or above and S-CVA/Ave of 0.90 or above were the minimum acceptable indices of items were used [30]. Items that were not achieving the minimum acceptable indices were revised and re-evaluated.

A pilot test was conducted and used to understand how respondents perceive and interpret the questions, thus helping in the identification of problematic questions that may cause response errors [31].

CFA was computed to understand how the tool was structured in the local context. Before conducting factor analysis, the following criteria were considered: The Kaiser–Meyer–Olkin Measure (KMO) was calculated and used for sampling adequacy thus, exceeding the threshold of KMO of 0.60. The Bartlett’s Test of Sphericity was also computed and used p < 0.01, confirmed the data set was appropriate for factor analysis for all the subscales of the tool [32].

The goodness of fit of the model was checked using chi-square statistics and maximum likelihood estimation. A small value indicated a good fit. Furthermore, a CFI parameter was also used to measure the improvement in model fitness, with a higher CFI indicating a better fit. A CFI value above 0.95 was an excellent fit, and 0.90 was an acceptable fit [33]. Moreover, similar to CFI, a TLI of higher value indicated more improvement from the null model. A value of above 0.95 was considered as an excellent fit, and 0.90 was considered an acceptable fit [33]. A small value indicated a good fit [33].

RMSEA was computed to indicate a degree of deviation from the null hypothesis. The result showed smaller values, indicating a better fit of the model. RMSEA showed a good fit and 0.08 value cut-points for an acceptable fit [33]. SRMR was performed for model fitness and showed a standardized measure of discrepancy between the data covariance matrix and the reproduced covariance matrix. A SRMR value of 0.10 or smaller indicated a good fit [33]. The Akaike Information Criterion (AIC) and Bayesian information criterion (BIC) were used for model comparison.

Factor analysis was computed and used to examine the psychometric properties of the tool, using EFA with Promax with Kaiser Normalization. EFA was conducted and used to explore the factor structure of the various constructs within the tool. EFA facilitated the assessment of the convergent validity of the emergent scale [34].

The principal Axis Factoring method of extraction, with Promax rotation, was calculated and used to determine the factor loadings, assess the validity, and provide a basis for the deletion of the item in the tool with poor factor loadings below 0.3 [35]. The decision made on the number of factors was extracted based on extracting factors that had an eigenvalue > 1 and the visual examination of the scree plot [35].

The tool's reliability was determined by the extent to which it performed consistently over repeated use. Internal consistency (Cronbach’s alpha > 0.70) was considered and described the extent to which all the items in a tool measure the same concept and connected to the inter-relatedness of the items [32]. Inter-rater reliability was computed. The two raters agreed with one another 80% or above, showing the amount of random measurement error was acceptable [32].

Test–retest reliability was calculated using (ICC), meaning the measurement tool was administered again to the same participants two weeks later [36]. This helped to assess the stability of the tool over time [36]. Oblique Promax rotation was used, which was an extension of Varimax rotation, to achieve a simple structure of factor loadings by allowing no orthogonal axes [34]. A factor loading of 0.32 and above was given 10% of the overlapping variance [36]. Multicollinearity was checked using a determinant score above the rule of thumb of 0.00001 [34]. Communality was calculated.

## Results

### Socio-demographic and economic characteristics of women with severe preeclampsia

Of the 264 study participants, 252 (95.5%) of them were married, 114 (43.2%) attended primary education, and 123 (46.6%) were between 15–24 years old (see Additional file 1: Table S1).

### Obstetric characteristics of the women with severe preeclampsia

Of 264 study participants, nearly half (45.8%) of neonates were male, and the majority (238, 90.2%) of neonates were singletons. More than three-fourths, 221 (83.7%), gave birth using normal vaginal delivery. Most mothers gave birth at term, 153 (58%), and 350 (94.7%) of them had severe preeclampsia. Of these, 136 (51.5%) had severe symptoms that persisted (see Additional file 1: Table S2).

### Face and content validation

There was an agreement between the seven experts that all the items in the tools were applicable for the current study setting. The item‑level content validity ranged from 0.86 to 1.00; the scale‑level content validity index was 0.97. This also indicated adequate content of each item in the four domains of the tool. None of the items were rejected by the experts, but they were suggested to change the wording of a few items. For example, item five was rephrased to enhance its face and content validity. Rewording of such items was computed to remove any ambiguous phrasing and promote an easier understanding of the item's layout, clarity, and comprehensiveness. All the items in the tool were required for domain coverage. After rephrasing the tool based on the feedback from the expert panel, the tool was then further tested in a pilot study.

### Pilot testing

In step 6 of the pre-test, the participants identified two items out of the 26 that needed to be rephrased due to lack of clarity. The rephrased version of the tool was tested in phase two of the QOL of women with severe preeclampsia. With pre-testing of the rephrased items, no further problems were identified with any of the items in the WHOQOL-BREF tool. Overall responses from all the participants were that the survey tool was interested, easy to read, understand, and complete. The team also noted the time taken to complete the tool by each woman with preeclampsia was approximately 15–20 min.

### Acceptability of local Sidamic version of the WHOQOL-BREF tool

All participants responded to all items in the local Sidamic WHOQOL-BREF version and marked them correctly. No missing items were found. Data collectors have reported no difficulties in asking the questions, and no participants have reported having any problems understanding the items. The average time taken to complete the scale was 17 min.

### Test–retest

The tool was tested for the second time two weeks later after the first measurement. A total of 74 women with severe preeclampsia were selected randomly to fill out the tools. The two-week test–retest reliability result was shown to have an excellent correlation between reliable strategy to assess these point scores (ICC for agreement 0.78; p < 0.001).

### Model fitness indices of confirmatory factor analysis of the WHOQOL-BREF tool

CFA was computed and used to confirm the hypotheses. It was using path analysis diagram to show variables in each domain. A standardized factor loading for each variable ranged from 0.97 to 0.99. CFA was used to test the fit of the structure, comparing the original English structure with the new structure. In CFA, the model fit indices were unacceptable (CFI = 0.87, RMSEA = 0.23, SRMR = 0.38, and TLI = 0.85) and pclose = 0.00 (see Additional file 1: Table S3).

### Bartlett’s Test of Sphericity and Kaiser–Meyer–Olkin Measure (KMO)

**Fig. 1:**
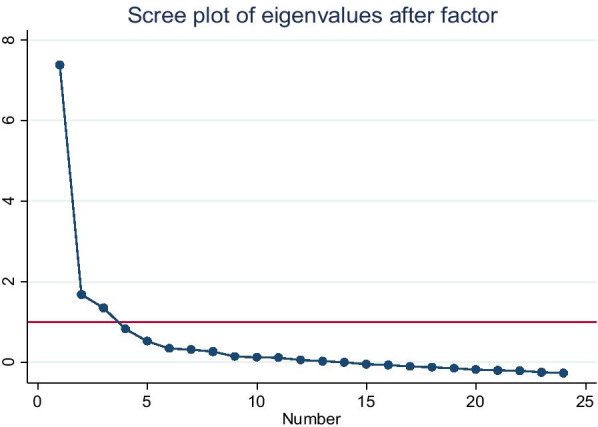
Graph of scree test and eigenvalues used to determine the number of factors extracted in southern Ethiopia 2019

The Kaiser–Meyer–Olkin Measure (KMO) of sampling adequacy was 0.96, and Bartlett’s Test of Sphericity p < 0.01, which confirmed that the data set was appropriate for conducting factor analysis of all the subscales (see Additional file 1: Table S4). EFA confirmed a three-factor structure was extracted, inconsistent with the original English structure (Fig. 1).

### Exploratory factor analysis

The WHOQOL-BREF tool factor structure was established and used principal factor using Promax rotation carried for the 26-items to determine factor loadings, reliability, and validity and remove any item. Internal consistency reliability Cronbach’s alpha was 0.98. Corrected item-total correlation means the correlation of the items designated with the summated score for all remaining items and ranges from 0.24 to 0.98. Alpha if item deleted means the Cronbach’s alpha reliability coefficient if the individual item was rejected from the tool ranges from 0.91 to 0.99. The result of the determinant score was 2.65 (see Additional file 1: Table S4).

### Reliability

Cronbach’s alpha was calculated using all 264 women with severe preeclampsia to determine the internal consistencies of the total items of the tool. The internal consistency reliabilities ranged from 0.8045 to 0.9123 indicating good-to-excellent reliability. All of the extracted factors showed acceptable Cronbach’s alpha (Table 1).

**Table 1:** Exploratory factor analysis with factor loadings of the Sidamic local language among women with severe preeclampsia quality of life tool in southern Ethiopia 2019

Items	Item description	Factor 1	Factor 2	Factor 3
Item 1	To what extent do you feel that physical pain prevents you from doing what you need to do?	− 0.1825	**0.6244**	**0.3137**
Item 2	How much do you need any medical treatment to function in your daily life?	− 0.1988	**0.6654**	0.2463
Item 3	How well are you to be able to get around?	**0.3432**	− 0.1618	0.0715
Item 4	Do you have enough energy for everyday life?	**0.5883**	− **0.3766**	0.0237
Item 5	How satisfied are you with your sleep?	**0.6011**	− 0.2230	0.1348
Item 6	How satisfied are you with your ability to perform your daily living activities?	**0.7004**	− 0.1507	0.2509
Item 7	How satisfied are you with your capacity to work?	**0.6314**	− 0.0768	0.2513
Item 8	How much do you enjoy life?	**0.6584**	− 0.1192	0.0191
Item 9	To what extent do you feel your life to be meaningful?	**0.5917**	− 0.1041	0.0891
Item 10	How well are you able to concentrate?	**0.6188**	− 0.0227	**0.3082**
Item 11	Are you able to accept your bodily appearance?	**0.4836**	0.2362	0.2836
Item 12	How satisfied are you with yourself?	**0.5941**	0.2154	0.2162
Item 13	How often do you have negative feelings, such as blue mood, despair, anxiety, depression?	− **0.4500**	0.1514	− 0.1476
Item 14	How satisfied are you with your personal relationships?	**0.5502**	0.1579	0.1581
Item 15	How satisfied are you with your sex life?	**0.5165**	0.2007	0.0727
Item 16	How satisfied are you with the support you get from your friends?	**0.6708**	0.1350	− 0.0236
Item 17	How safe do you feel in your daily life?	**0.6244**	0.0396	0.0585
Item 18	How healthy is your physical environment?	**0.5645**	− 0.1076	− 0.0102
Item 19	Have you enough money to meet your needs?	**0.6752**	− 0.0326	− 0.2978
Item 20	How available to you is the information that you need in your day-to-day life?	**0.5844**	0.2359	− **0.4290**
Item 21	To what extent do you have the opportunity for leisure activities?	**0.4000**	**0.3933**	**0.3583**
Item 22	How satisfied are you with the conditions of your living place?	**0.5288**	0.0091	− 0.1066
Item 23	How satisfied are you with your access to health services?	**0.5695**	**0.3802**	− 0.2179
Item 24	How satisfied are you with your mode of transportation?	**0.5674**	0.0914	− s**0.5284**
	Cronbach’s’ alpha	0.9123	0.8045	0.8151

Items with high loading (> 0.32) to factors were indicated in bold Extraction Method: Principal Axis Factoring Rotation Method: Promax with Kaiser Normalization

### Validity

The previously validated and widely used WHOQOL-BREF tool was shown to have four-factor domains. However, after a Promax rotation, we extracted three new factor structures using EFA for current research. The extracted new factor structures explained the total variance of 91%. Therefore, the factor analysis was repeated for the second time, using an eigenvalue greater than one; it was forced to extract three new factors. There was no cross-loading of any item among the three factors. Twenty-two items were loaded most strongly on Factor one. Five items were loaded most strongly onto Factor two and Factor three. The names of the three new factor structures were Factor one (Item 3–24), Factor two (Item 1, 2, 4, 21, 23), and Factor three (Item 1, 10, 20, 21, 24). The items that were loaded onto each of the three factors were shown (Table 1).

## Discussion

The failure of the original scale in this study population highlights the importance of culturally adapting the tool to local settings to obtain accurate and reliable estimates. The reason for the failure of the original scale in this setting, there is an overtime change in a society in the perception of the local context and cultural perspectives [24]. This culturally adapted generic tool failed the assumptions because it did not measure the same concepts in the original and target settings [22, 24].

The WHOQOL-BREF tool was assessed to adequately measure what it intends to measure and whether the questions are relevant and clear. This is supported by other studies [22]. The content of the tool was conceptually grounded in the different works of literature. A panel of experts was used to assess the face and content validity of the measure. The consensus among the seven experts was that all the items in the tool supported the content validity of the WHOQOL-BREF tool [22]. The comprehensive process of tool translation and cultural adaption was followed for each item to the target culture while retaining the meaning and intent of the original items.

Forward translators from the source to the target language were conducted using people fluent in both languages and cultural perspectives. This is suggested by other studies [16, 37]. This might be due to the study team's suggested meeting between forwarding translators held in order to identify a problem in wording, differences, and discrepancies between the two translated versions and any differences through discussion and consensus.

Two back-translated versions of the tool were compared. This has been supported by other studies [17, 18]. We followed the instructions, items, and response format of the original tool in the source language wording and grammatical structure of the sentences. Similarity in meaning and relevance were considered. Any ambiguities and discrepancies in cultural meaning, and instructions, items, response format, between the two back-translations, and between each one of the two back-translations and the original tool in the source language were discussed. Raised issues were resolved through consensus among the committee members to derive a pre-final version of the tool in the target language [17, 18].

Translated and back-translated versions were followed by the same validation process, evaluating and repeating until no ambiguities or discrepancies were found. This has been supported by other studies [21, 22]. The role of the committees is to evaluate, revise, and combine the instructions, items, and response format of the back-translated tool. It has confirmed conceptual, semantic, and content equivalency, and it is developing the pre-final version of the target language for pilot and psychometric testing.

A pilot study was computed using a series of cognitive interviews with women with severe preeclampsia revealed that the questionnaire was easy to read, understand and use. This is suggested by other studies [20, 31]. They ensured the tool administration's validity, acceptability, and feasibility. This might be due to each patient being asked to rate the instructions and items of the tool, provide suggestions on how to rewrite the statements to make the language more clear. At least 20% of the sample was considered and re-evaluated [20, 31].

A minimum inter-rater agreement was calculated among the sample and found to be 80%. Conceptual, semantic, and content equivalence of the translated tool were considered. This is suggested by other studies [32, 36]. This might further improve the structure of sentences used in the instructions and items of the pre-final version of the local Sidamigna language and allow the target women with severe preeclampsia to easily understand the language before the actual psychometric test begins.

A test–retest assessment was conducted and provided evidence for the stability of the tool over time. This is agreed with other studies [34, 36]. This was because Cronbach’s alpha provided an estimate of the internal consistency. However, it did not show the stability of the test over time, which was better, estimated using the test–retest reliability method. This was also used to decide what time points to use in the data analysis [36]. This finding is comparable with the English validation study, ensuring that responses are not too varied across the time [36]. The measurement taken at any point in time using the Sidamic QoL tool is reliable. This may also inspire researchers in the future to interpret their results from the Sidamic version of the tool. Conceptual and content equivalence of the items of the pre-final version of the local language tool was computed using an expert panel. This is supported by other studies [34, 37]. The reasons for this included instructions, the response format, and the items of the tool are evaluated for conceptual equivalence by seven members of an expert panel. They are knowledgeable about the content areas of the tool, the target population in which the tool is used, and speak Sidamigna. They stress problematic words or items, and it is easier to detect any discrepancies in the tool if the participants were interviewed face-to-face.

A psychometric test of the pre-final version of the translated tool was calculated. The result of Bartlett’s Test of Sphericity was (p < 0.01), and KMO was 0.96. This confirmed that our data were appropriate for EFA and revealed pattern relationships between the variables. As this requirement was met, it was confirmed that distinct and reliable factors were produced [32].

Factor analysis was computed and used to identify the new meaningful underlying variables in each factor domain. This is supported by another study [34]. We considered the maximum likelihood and principal axis factoring methods for factor extraction. The factor loadings were determined to determine whether any item was removed if factor loadings were below 0.32 [34]. This is due to factors extracted successively until a large enough variance is accounted for.

Promax rotation with the Kaiser Normalization method for factor extraction was computed. This has been suggested by other studies [35, 36]. A possible explanation might be using Promax rotation in a scale construction context provided a simple structure that is desirable for an EFA.

Rotated factor loadings, eigenvalues > 1, and scree tests are used for the interpretation of factor analysis. This has been suggested by another study [35]. The Scree test in combination with the eigenvalues was used to determine the number of factors retained. Eigenvalue decided the number of factors and showed the amount of information represented by a common factor among a set of analyzed variables, where a higher value indicated more information. The number of eigenvalues 1.0 or greater was counted and used to decide the number of factors. The standardized factor loading for each variable is greater than 97%. This finding is similar to that of another study [35]. This could be because the measured variable contributes to the factor; thus, high factor standardized loading scores indicate that the variables better account for the dimensions of the factors [35].

Factor loading for a variable 0.32 and above was considered to resolve the issue of a non-significant loading item. This is agreed with another study [34]. A high factor loading score indicates the dimensions of the factors better account for and represents the strength of the correlation between the variable and the factor. A variable below 0.32 is considered as a weak relationship between the variables not considered in factor construction [34].

The internal consistency reliabilities ranged from 0.8045 to 0.9123 indicating good-to-excellent reliability. This is supported by other studies [18, 32]. This indicated the good-to-excellent internal consistency and reliability of the tool. A possible explanation might be the reliability of the value of alpha-if-item deleted data useful, as they display the effect on the total scale score reliability of removing any single item from the tool. Items that were removed enhance subscale reliability from the scale and strengthen internal consistency by removing any weak items.

The extracted new factor structure is inconsistent with the factor structure reported in the original English version (WHOQOL-BREF tool). This is supported by other studies [18, 24]. This is due to previously validated tools not always being translated properly and does not necessarily mean they are valid in other settings, cultures, or contexts, possibly due to poor translation. Findings based on such instruments may not accurately reflect what they are intended to measure.

The determinant score was 2.65. This finding is shown above the rule of thumb of 0.00001 as it confirms no multicollinearity among variables. This is supported by another study [34]. This might be due to the determinant score being significantly different from zero, which indicates an absence of multicollinearity and patterned relationships among the variables.

Communality among variables was greater than 78%. This finding is similar to other studies [35, 37]. This is due to a particular set of factors that explained a lot of the variance of a variable, and the factor analysis explained the variances through the common factors shared.

## Implications for policy and clinical practice

Findings from this cultural adaptation and validation of the QoL tool may have clinical implications for physicians, gynecologists, and other health care professionals who provide care for women with severe preeclampsia. Providing clinicians and researchers with access to a reliable and valid tool could enhance the delivery of quality of care for women with severe preeclampsia. Quality of care will depend on the accurate assessment and understanding of an individual’s cultural, linguistic, and ethnic background to improve responses to clinical problems [24].

## Limitations and strengths of the study

In this study, we used a cross-sectional study design that could not determine causality; temporal sequence between exposure and disease could not be established.

The other limitation was the possibility of recall bias. The interval of two years on average between the index-pregnancy and the time of the survey may have affected the results. However, pregnancy is a unique time in the life of any woman, and recall of pregnancy-related events in women even 30 years or more after delivery is reproducible and reasonably accurate.

A strength of this study included conceptual equivalence between the source and target languages. Evaluating and clarifying the concepts in each item of the original and target tool for current research was achieved. Qualified translators and committee members were considered to enhance the quality of the forward translation, back-translation, and transcultural validation of the tool. Forward translators were selected, certified in the local language, and have in-depth experience in the culture of the target language.

## Conclusions

The failure of the original scale in this study population highlights the importance of culturally adapting tool to local settings. EFA confirmed a three-factor structure, inconsistent with the original English structure.

## Supplementary Information

**Additional file 1.** The translation process, pilot test, Table S1, Table S2, Table S3 and Table S4 in Sidama zone, southern Ethiopia 2019.

## Data Availability

The data that support the findings of this study is available from the corresponding author upon reasonable request in the form of SPSS Version 23 and STATA Version 14.

## Abbreviations

AIC: Akaike’s information criteria
BIC: Bayesian information criteria
CFI: Comparative fit index
CVI: Content validity of individual items
ICC: Intraclass correlation coefficient
RMSEA: Root mean square error of approximation
SRMR: Standardized root mean square residual
S-CVI: Scale’s content validity index
TLI: Tucker Lewis index
