# Supplementary material for: Cultural adaptation and validation of the Sidamic version of the World Health Organization Quality-of-Life-Bref Scale measuring the quality of life of women with severe preeclampsia in southern Ethiopia, 2020
Source: Health Qual Life Outcomes. 2021 Oct 12;19:239. doi: 10.1186/s12955-021-01872-z (PMC8513212; doi:10.1186/s12955-021-01872-z)
Supplement: Supplementary file 1 — Additional file 1. The translation process, pilot test, Table S1, Table S2, Table S3 and Table S4 in Sidama zone, southern Ethiopia 2019. [file 12955_2021_1872_MOESM1_ESM.docx]

Supplementary Materials

**Methods**

**Translation process and pilot test**

**Step 1: Mapping the available scales for this purpose**

A WHOQOL-BREF tool was chosen. The tool provided an overall score and a score by domain for quality of life measurement. It consisted of 26 items across 4 domains: physical (7items), psychological (6items), social relationship (3items) and environment (8items) used. There were two global scores of overall QOL (1item) and overall satisfaction with health (1item). The WHOQOL-BREF tool was used Likert-type response options which help respondents express their true feelings or thoughts in more detail (1). A wider response range was chosen to enhance scale reliability (1). It was contained five Likert response scales: “1=very poor/very dissatisfied/not at all”, “2=poor/dissatisfied/a little”, “3=neither poor nor good/neither satisfied nor dissatisfied/a moderate amount”, 4=good/satisfied/very much” and“5=very good/very satisfied/an extreme amount”. Each domain was made of questions for which the scores varied between one and five (2). The overall quality of life and general health items were not included in the calculation of domain scores (2). WHOQOL-BREF tool was obtained using a free of charge and provided their contact of the national WHOQOL center for permission (3).

**Step 2: Face, content validation, and expert panel discussion**

Face and content validation of the tool through four domains of the WHOQOL-BREF tool by the expert panel with four midwife experts, epidemiologists, gynecologists, and bilingual backward translators were performed. In relation to face validation, the panel of experts independently assessed the WHOQOL-BREF tool for readability, understanding, clarity, and ease of use (4). The expert panel discussion helped to resolve inconsistencies and finalize the scale for pretesting. Decisions were made by this committee considered equivalence between the source and target version and examined the source and back-translated questionnaires for all such equivalences (5). A consensus was reached on the items, instructions, and response options.

**Step 3: Forward translation and synthesis by an expert panel**

Two bilinguals (in Sidamic and English language) forward translators who were capable to translate the original (English) version into the Sidamic version were selected. The first translator had a background of clinical perspective. The other translator had no clinical background and was considered as a naive translator and more likely to detect the different meaning of the original than the first translator. Translations into the Sidamic language were more accurately reflected the tones of the language. The translations were compared and discrepancies were noted during the translation process. The poorer wording choices were identified and resolved in a discussion between the translators (6). The translators produced a document of the translation including the required steps of translations. Items, contents, response options, and instructions of the questionnaires were translated in this way (5).

**Step 4: Synthesis of the forward translations**

The team of experts and two translators discussed the discrepancies and synthesized the results of the translations. Then, one common document (T-12) was produced from the first translator’s (T1) and the second translator’s (T2).

**Step 5: Back translation and Expert Panel Review**

We worked on the T-12 version of the scale and were given for two bilinguals (in Sidamic and English language) who were blinded to the original version (7). The back translations (BT1 and BT2) were performed by two translators with the source language (English). Furthermore, the two translators were neither aware nor informed clarifying the concepts investigated by each item, and both of them were non-medical in the profession. The main reasons for this included: to avoid information bias and unexpected meanings of the items in the translated scale (5, 7). This was a validity checking process to ensure that the translated version was reflected the same item content as the original versions. Moreover, this step was helped to clarify wording in the translations and assured a consistent translation as well.

**Step 6: Pre-test**

A pre-test was conducted among 62 severe preeclamptic women aimed to test its consistency before actual data collection. Sample size determination for this pre-test was based on recommendations for factor analysis no fewer than 50 and no more than 100 (8). Each participant was interviewed to investigate how each questionnaire item and response was organized. Both the meaning of the items and responses explored and described a successful adaptation process in the local language (8).

**Submission of documentation**

This stage was considered the submission of all the reports and forms to the committee kept track of the translated version. It also assured whether or not the recommended steps were followed in the adaptation process.

**Results**

**Table S1: Socio-demographic and economic characteristics of pregnant women with severe preeclampsia in Sidama zone, southern Ethiopia 2019**

| Variables | Frequency (n) | Percentages (%) |
| --- | --- | --- |
| Age of mother [year] |  |  |
| 15-24 | 123 | 46.6 |
| 25-34 | 122 | 46.2 |
| ≥35 | 19 | 7.2 |
| Maternal education |  |  |
| No formal education | 49 | 18.6 |
| Primary education | 114 | 43.2 |
| Secondary education | 80 | 30.3 |
| College/University | 21 | 8 |
| Husband education |  |  |
| No formal education | 28 | 10.6 |
| Primary education | 77 | 29.2 |
| Secondary education | 92 | 34.8 |
| College/university | 61 | 23.1 |
| Others (no known husband) | 6 | 2.3 |
| Religion |  |  |
| Orthodox | 23 | 8.7 |
| Protestant | 213 | 80.7 |
| Muslim | 15 | 5.7 |
| Others(Catholic, nonbelievers) | 13 | 4.9 |
| Maternal occupation |  |  |
| House wife | 152 | 57.6 |
| Merchant | 32 | 12.1 |
| Employed | 23 | 8.7 |
| Farmer | 2 | 0.8 |
| Daily laborer | 13 | 4.9 |
| Student | 42 | 15.9 |
| Husband occupation |  |  |
| Student | 18 | 6.8 |
| Unemployed | 2 | 0.8 |
| Merchant | 88 | 33.3 |
| Employed | 63 | 23.9 |
| Farmer | 52 | 19.7 |
| Daily laborer | 40 | 15.2 |
| Place of residence |  |  |
| Urban | 70 | 26.5 |
| Rural | 194 | 73.5 |

**Table S2: Obstetric characteristics of the women with severe preeclampsia in Sidama zone, southern Ethiopia 2019**

| Variables | Frequency (n) | Percentage (%) |
| --- | --- | --- |
| Fetal sex |  |  |
| Male | 121 | 45.8 |
| Female | 103 | 39 |
| Not applicable (abortion) | 40 | 15.2 |
| Number of fetus |  |  |
| Singleton | 238 | 90.2 |
| Twin | 26 | 9.9 |
| Parity |  |  |
| 1 | 131 | 49.6 |
| 2-4 | 108 | 40.9 |
| ≥5 | 25 | 9.5 |
| Gravidity |  |  |
| 1 | 129 | 48.9 |
| 2-4 | 104 | 39.4 |
| ≥5 | 31 | 11.7 |
| Mode of delivery |  |  |
| SVD | 221 | 83.7 |
| Vacuum assisted delivery | 131 | 49.6 |
| Cesarean delivery | 32 | 12.1 |
| Forceps delivery | 9 | 3.4 |
| Others(destructive vaginal delivery) | 4 | 1.5 |
| Reasons for termination of pregnancy |  |  |
| Term pregnancy | 153 | 58 |
| Persistence of severity symptoms | 136 | 51.5 |
| Intrauterine fetal death | 28 | 10.6 |
| Twin pregnancy | 14 | 5.3 |
| Admission to maternal ICU | 13 | 4.9 |
| Active bleeding | 19 | 7.2 |
| Organ function derangement | 17 | 6.4 |
| Fetal growth restriction | 36 | 13.6 |
| Evidence of non-reassuring fetal condition | 22 | 8.3 |
| Eclampsia | 39 | 14.8 |

SVD- Spontaneous Vaginal Delivery, ICU-Intensive Care Unit

**Table S3: Model fitness indices fitting types of factor in confirmatory factor analysis for 24-item locally WHOQOL_BREF scale in Sidama zone, southern Ethiopia 2019**

| Fit indices statistics | Values | Description |
| --- | --- | --- |
| Likelihood Ratio Test  χ2_ms(246)  P> χ2  χ2_bs(276)  P> χ2 | 3644.95  p-value<0.001  27314.780  p-value<0.001 | Model vs. saturated  Baseline vs. saturated |
| Population Error  RMSEA  90% CI, lower bound  upper bound  pclose | 0.23  0.22  0.24  p-value<0.001 | Root mean squared error of approximation  Probability RMSEA <= 0.05 |
| Information Criteria  AIC  BIC | -1521.90  -1242.98 | Akaike’s Information Criteria  Bayesian Information Criteria |
| Baseline comparison  CFI  TLI | 0.87  0.85 | Comparative fit index  Tucker-Lewis index |
| Size of residuals  SRMR  CD | 0.38  1.000 | Standardized root mean squared residual  Coefficient of determination |

**Table S4: Internal consistencies, item-total correlations, and alpha if item deleted from the Sidamic version WHOQOL BREF tool quality of Life of women with severe preeclampsia Sidama zone, southern Ethiopia 2019**

| Items | Item description | Corrected item-total  correlation | Alpha if item  deleted |
| --- | --- | --- | --- |
|  | **Overall quality of life and your health** |  |  |
| Item 1 | How would you rate your quality of life? | 0.36 | 0.96 |
| Item 2 | How satisfied are you with your health? | 0.24 | 0.95 |
|  | **Domain 1: Physical** |  |  |
| Item 3 | To what extent do you feel that physical pain prevents you from doing what you need to do? | 0.84 | 0.97 |
| Item 4 | How much do you need any medical treatment to function in your daily life? | 0.89 | 0.95 |
| Item 5 | How well are you to be able to get around? | 0.80 | 0.98 |
| Item 6 | Do you have enough energy for everyday life? | 0.94 | 0.99 |
| Item 7 | How satisfied are you with your sleep? | 0.88 | 0.99 |
| Item 8 | How satisfied are you with your ability to perform your daily living activities? | 0.86 | 0.96 |
| Item 9 | How satisfied are you with your capacity to work? | 0.83 | 0.98 |
|  | **Domain 2: Psychological** |  |  |
| Item 10 | How much do you enjoy life? | 0.77 | 0.99 |
| Item 11 | To what extent do you feel your life to be meaningful? | 0.76 | 0.99 |
| Item 12 | How well are you able to concentrate? | 0.79 | 0.99 |
| Item 13 | Are you able to accept your bodily appearance? | 0.92 | 0.96 |
| Item 14 | How satisfied are you with yourself? | 0.81 | 0.97 |
| Item 15 | How often do you have negative feelings, such as blue mood, despair, anxiety, depression? | 0.93 | 0.98 |
|  | **Domain 3: Social relationship** |  |  |
| Item 16 | How satisfied are you with your personal relationships? | 0.85 | 0.97 |
| Item 17 | How satisfied are you with your sex life? | 0.90 | 0.95 |
| Item 18 | How satisfied are you with the support you get from your friends? | 0.86 | 0.96 |
|  | **Domain 4: Environment** |  |  |
| Item 19 | How safe do you feel in your daily life? | 0.88 | 0.99 |
| Item 20 | How healthy is your physical environment? | 0.78 | 0.99 |
| Item 21 | Have you enough money to meet your needs? | 0.93 | 0.98 |
| Item 22 | How available to you is the information that you need in your day-to-day life? | 0.89 | 0.95 |
| Item 23 | To what extent do you have the opportunity for leisure activities? | 0.98 | 0.91 |
| Item 24 | How satisfied are you with the conditions of your living place? | 0.76 | 0.95 |
| Item 25 | How satisfied are you with your access to health services? | 0.79 | 0.96 |
| Item 26 | How satisfied are you with your mode of transportation? | 0.88 | 0.97 |
|  | Overall Cronbach’s alpha (26 items) |  | 0.98 |
|  | Standardized item alpha |  | 0.98 |
|  | Kaiser-Meyer-Olkin Measure of Sampling Adequacy |  | 0.96 |
|  | Bartlett's Test of Sphericity Approx. Chi-Square  df.  Sig. |  | 26297.37  276  0.000 |
|  | Correlation matrix: determinant score test |  | 2.65 |
|  | Communality |  | 0.78 |

Sig. Significance, df-degree of freedom

**References**

1. Tim C et al. Development, reliability, and validity of the Chichewa WHOQOL-BREF in adults in Lilongwe, Malawi. BMC Research Notes2012; 5(346)

2. Cristina R et al. Development and validation of the Somali WHOQOL-BREF among refugees living in the USA. Qual Life Res 2015); 24:1503-13

3. Wan Ho et al. Reliability and Validity of the Korean World Health Organization Quality of Life (WHOQOL)-BREF in People with Physical Impairments. Ann Rehabil Med 2013; 37 (4):488-97

4. Mary H et al. Validation of the WHOQOL-Bref: psychometric properties and normative data for the Norwegian general population. Health Qual Life Outcomes 2021; 19 (13)

5. Gholami A et al. Application of WHOQOL-BREF in measuring quality of life in health-care staff. Int J Prev Med 2013; 4:809-817

6. Chi-Wen C et al. Development and validation of a WHOQOL-BREF Taiwanese audio player-assisted interview version for the elderly who use a spoken dialect. Qual Life Res2007; 16:1375-81

7. Li Gholami et al. Application of the World Health Organization Quality of Life Instrument, Short Form (WHOQOL-BREF) to patients with cataracts. Epidemiology and Health2016;38 (e2016005):7

8. Linn G et al. Cross-cultural adaptation of research instruments: language, setting, time and statistical considerations. Gjersinget al BMC Medical Research Methodology2010; 10 (13)
